# Integrating evolution into medical education for women’s health care practitioners

**DOI:** 10.1093/emph/eoaa009

**Published:** 2020-04-06

**Authors:** Michael L Power, Carrie Snead, Eda G Reed, Jay Schulkin

**Affiliations:** e1 Smithsonian National Zoological Park and Conservation Biology Institute, Washington, DC 20013-7012, USA; e2 American College of Obstetricians and Gynecologists, Washington, DC 20024-2188, USA; e3 Johns Hopkins University, Baltimore, MD 21205, USA; e4 Obstetrics & Gynecology, University of Washington School of Medicine, Seattle, WA 98195, USA

**Keywords:** lactation, microbiome, medical training, obstetrician–gynecologists

## Abstract

Evolution is a fundamental principle in biology; however, it has been neglected in medical education. We argue that an evolutionary perspective is especially important for women’s health care providers, as selection will act strongly on reproductive parameters, and the biological costs of female reproduction are generally more resource expensive than for men (e.g. due to gestation and lactation) with greater effects on health and wellbeing. An evolutionary perspective is needed to understand antibiotic resistance, disease and health risks associated with mismatches between our evolved adaptations and current conditions, the importance of the microbiome and the maternal role in how infants acquire and develop their early-life microbiome (vaginal birth, lactation), and the importance of breastmilk as a biochemical signal from mothers to their babies. We present data that obstetrician–gynecologists’ views regarding the inclusion of evolution within their training is generally positive, but many barriers are perceived. Requiring coursework in evolutionary biology with an emphasis on evolutionary medicine prior to enrollment in medical school may be a solution.

## Introduction

*No biological problem is solved until both the proximate and the evolutionary causation has been elucidated. Furthermore, the study of evolutionary causes is as legitimate a part of biology as is the study of the usually physicochemical proximate causes.*

E. Mayr, 1982 [1]

*Nothing in biology makes sense except in the light of evolution*.

Theodosius Dobzhansky, 1973 [2]

Medical science is inherently a biological science. A firm understanding of biology is necessary to engage in medical science, medical research and, to a lesser but still significant extent, to practice clinical medicine. Evolution is recognized as a fundamental organizing principle of modern biology, as illustrated in the epigraphs of this commentary. Over the past century, the field of evolutionary biology became an established and productive perspective in the biological science community, and yet it can be argued that evolution is the area of biology that is the least well-integrated into medical education [3, 4]. Medical education currently focuses on proximate explanations of biological principles and mechanisms important to human health and disease and does not always include ultimate or evolutionary explanations [5]. Proximate explanations are vital to understanding medical conditions and treating and preventing diseases, but they are insufficient in answering the ‘why’ questions posed both by medical students and researchers. An evolutionary perspective enables a deeper understanding of biological phenomena and can even predict them. For example, microbial antibiotic resistance and its frightening spread are understandable and predictable from an evolutionary perspective. Accordingly, our medical and public health efforts to slow the increase in antibiotic resistant pathogens would be enhanced by taking evolution into account [6].

We argue that an evolutionary perspective is particularly relevant to the practice of obstetrics and gynecology. Reproduction is under strong selective pressure, and women’s reproductive efforts (gestation and lactation) are more complex, resource intensive and prone to complications that can affect health and wellbeing compared to those of men. For example, after implantation of the human blastocyst, the placental villi invade the maternal endometrium. Abnormal placentation is associated with potentially life-threatening (for mother and/or fetus) pregnancy complications, including preeclampsia, hemorrhage and preterm birth [7]. In a normal pregnancy, the invasion of the decidua by placental villi only intrudes into the decidual basalis, but in a small proportion of pregnancies (0.2–0.4%; [8]) the villi invade the myometrium (placenta accrete, increta or percreta, depending on the extent of the invasion of the myometrium). This can lead to life-threatening hemorrhaging after birth [8]. Preeclampsia, which affects ∼4% of US pregnancies [9] and is the second leading cause of maternal mortality worldwide [9], is also a disease of abnormal placentation. In preeclampsia, a deficiency in the villi invasion and remodeling of the uterine spiral arteries results in inadequate placentation and a maternal inflammatory response [7].

Preeclampsia has been suggested to have an evolutionary origin in parent–offspring conflict [10, 11]. In brief, the highly invasive hemochorial human placenta allows the fetus to release hormones and other biologically active molecules into maternal circulation that affect maternal metabolism and physiology. From the evolutionary ‘perspective’ of the fetus, receiving maximal resources from the mother is beneficial even if it compromises her ability for future reproduction. In contrast, the reproductive fitness of the mother is enhanced if she only provides sufficient resources to her fetus while minimally affecting her future reproductive potential. Hence, an evolutionary conflict between mother and fetus [10]. Studies have shown that many of the genes associated with preeclampsia are under positive selective pressure [7] and that some of these genes are associated with placentation in species with a less invasive placenta type than human placenta [11, 12]. These studies provide supporting evidence for the proposed parent–offspring conflict model underlying preeclampsia [10].

Another important concept is that evolution results in adaptive solutions to past challenges and does not predict future circumstances. Are some of the complications and problems of childbirth we face today a result of a mismatch between our species’ evolved mechanisms for successful reproduction and modern clinical practice? Aspects of distinctly human evolution, such as obligate midwifery, should be considered in order to create effective and appropriate birthing practices [13, 14]. For example, delivery assistants historically had close emotional relationships with the mother, and continuous emotional support by a doula during labor significantly reduced the rate of cesarean sections in a US hospital [15]. Evolutionary knowledge incorporated into birth plans could aid physicians’ holistic treatment of patients [14].

Although the factual link between medicine and evolution exists, it is apparent that integrating evolutionary biology into medical education faces several roadblocks. The standard medical education is already replete with complex subjects, an ever-increasing list of new medical technologies and a long period of training [16]. To add in training on the evolutionary perspective to this curriculum would certainly appear daunting. Proponents could be accused of requiring one more piece of science to be stuffed into the minds of young physicians by medical educators who are not fully qualified to teach the subject. However, including the evolutionary perspective in medical education arms physicians with an understanding of the ultimate (evolutionary) answers to clinical questions, rather than simply proximate (basic mechanistic) explanations, providing physicians with a more complete understanding of the etiology of health and disease [1, 4, 17, 18].

There is support for the value of training in evolution for practicing obstetrician–gynecologists. In a 2017 study of practicing obstetrician–gynecologists we conducted, 600 Fellows of the American College of Obstetricians and Gynecologists were contacted to participate in a survey to assess their experiences and beliefs regarding education and training in evolution in medical schools. Of these 600 individuals, 229 returned surveys. Most respondents (64.4%) believed that it is either ‘important’ or ‘vital’ for medical students to have a basic understanding of evolutionary biology, and only 10% reported believing that it is not important. Responses were varied when determining how evolution education should be incorporated into medical school curriculum, but a majority does support incorporation in some fashion (Table 1). In addition, 81.1% agreed that residency programs should devote time and resources to exposing their residents to the tenets of evolutionary medicine either as an option (47.0%) or a requirement (34.1%).

**Table 1.** Respondent perspectives of incorporation of evolutionary education into medical school

	Absolutely not	Only with minimal disruption to existing curriculum	Yes, but must be balanced against the disruption to the existing curriculum	Absolutely yes
Required course(s) on the importance of evolutionary biology to medicine	31 (14.2%)	70 (32.1%)	82 (37.6%)	35 (16.1%)
Dedicated lectures within existing courses linking evolution to medicine	20 (9.2%)	74 (33.9%)	79 (36.2%)	45 (20.6%)
Optional course(s) on the evolutionary perspective and medicine	25 (11.5%)	65 (30.0%)	70 (32.3%)	57 (26.3%)
Assigned readings on the role of evolution in medicine	30 (13.7%)	82 (37.4%)	67 (30.6)	40 (18.3)
Guest lectures by evolutionary biologists	22 (10.1%)	87 (39.9%)	72 (33.0%)	37 (17.0%)

### Evolution, Public Health and the One Health Concept

An evolutionary perspective may be most appropriate in the field of public health where, instead of individuals, the focus is the overall health and disease risks of populations [6, 19]. More than half of the obstetrician–gynecologists responding to our survey (52.9%) indicated that an evolutionary perspective is important or very important for public health (Table 2). There are many public health issues with strong links to evolution. For example, given the relevance of climate change in the context of disease, most recently as a purported element in the spread of Zika virus [20], understanding the future implications of evolution in the field of medicine is vital to addressing emergent health care concerns [21].

**Table 2.** Relevance of an evolutionary perspective in various aspects of medicine

	Not important	Somewhat important	Important	Very important
Medical research	23 (10.4%)	60 (27.1%)	74 (33.5%)	64 (29.0%)
Medical education	29 (13.1%)	64 (29.0%)	76 (34.4%)	52 (23.5%)
Public health	33 (14.9%)	71 (32.1%)	63 (28.5%)	54 (24.4%)
Clinical medicine	36 (16.3%)	79 (35.7%)	64 (29.0%)	42 (19.0%)

Antimicrobial resistance is another public health challenge that has intrinsic evolutionary underpinnings. The rapid rise and broad extent of current antibiotic resistance is due to several factors, one intrinsic to bacteria (horizontal gene transfer) but the others are human factors. Horizontal gene transfer, the transfer of genetic material between different microbial taxa, greatly enhances the ability of resistance genes to spread. But, the primary culprit for the rapid spread of antibiotic resistance is the overuse and inappropriate use of antibiotics by people, especially the wide-spread use of antibiotics in the food animal business [22]. However, the advent of antibiotic resistance in bacteria is predicted to occur by evolutionary theory, even if antibiotics are used in judicious and appropriate ways. For example, the sexually transmitted disease gonorrhea used to be easily and quickly cured by penicillin. Now the only effective treatment is a two-drug cocktail of third-generation antibiotics [23], and there are worrying signs of resistance to these may be developing.

Regarding environmental public health, many modern disease risks are linked to a mismatch between the environment we evolved under and the one we have created, through both direct and indirect effects on human health. Air pollution is associated with increased risks of respiratory tract infections and cardiovascular disease [24, 25] and has also been shown to be associated with preterm birth and early miscarriage [26, 27]. The One Health concept integrates the essential connections between human health and disease and both animal and environmental health [28]. The combination of the degradation of the environment, spreading pandemics associated with increased human and domestic animal contact with wild animals (e.g. COVID-19), and climate change altering, and in many cases, expanding the geographic ranges of disease vectors, has led global public health to adopt the One Health concept. One Health is intrinsically evolutionary in its theory and practice.

### Evolution, the Microbiome, and Women’s Health

As our technology advances and our knowledge accumulates, more and more of the diseases and health risks humans face are shown to have an evolutionary component. A recent example of technological advance uncovering an important new area of health science research that we argue has a necessary evolutionary component is the microbiome. Below we discuss some areas where the evolutionary link in microbiome research may have significant impact on maternal, fetal and neonatal health and therefore are an important component of the required knowledge of women’s health providers.

A healthy vaginal microbiome confers many advantages, most of which are not fully understood. The study of the vaginal microbiome should inform and guide practice in areas such as bacterial vaginosis (BV) and other infections of the lower reproductive tract. BV, a common problem among women of child-bearing age with a US prevalence ranging from 6.1% to 51.4% in high-risk populations [29], is at its core a disease of microbiome dysbiosis. The normal vaginal microbial community has been disrupted leading to overgrowth of pathogenic or at least noncommensal microorganisms [30], which can increase the risks for health issues such as a woman’s vulnerable to sexually transmitted infections, pelvic inflammatory disease, and during pregnancy, premature birth and fetal loss [31]. Often used characteristics to screen for BV are a vaginal pH > 4.5 [32] and a low prevalence of *Lactobacillus* species among the vaginal microbiota [33]. However, these characteristics exist in asymptomatic healthy women, especially among women of African descent and of Hispanic origin [34]. Interestingly, the vaginal communities of asymptomatic women that contained few, if any, *Lactobacillus* species had other lactic acid-producing species, suggesting that the production of lactic acid may be the important functional role of the human vaginal microbiome [35, 36]. The human vaginal microbiome appears to be unique in being dominated by *Lactobacillus* or other lactic acid-producing species, as these are not found in the vaginal microbiomes of nonhuman primates including our closest relative, the chimpanzee [37]. Understanding the coevolution of the human vaginal microbiome is likely to produce important insights into vaginal and neonatal health.

As the baby goes through the birth canal, it becomes inoculated with maternal vaginal microbes that are the start of the neonate’s microbiome. The vertical transmission (mother-to-neonate) of a healthy microbiome has significant effects on the health and wellbeing of the neonate that last into adult life [38]. However, currently in the USA, 32% of deliveries are by cesarean section [39]. Those babies miss much of the initial inoculation by maternal microbes and differ in their early gut microbiome from that of infants born by vaginal delivery. Infants born by cesarean section are also at greater risk for later adverse conditions such as obesity and asthma and there are convincing links between these risks and the early life microbiome [40]. Pilot research on vaginal seeding has shown some promise [41] but has inherent risks as well [42]. A proportion of high-risk pregnancies will always necessitate cesarean delivery, but an evolutionary perspective (and the expanding work on microbiomes) suggests that women (and providers) should be informed of the potential risks to their infant’s later health due to elective cesarean.

The human neonate is born with an immature, adaptive immune system; it is protected by maternal Immunoglobulin G that crosses the placenta by an active transport system that results in fetal blood concentration of IgG being higher that maternal blood [43]. After birth, if breastfed, the human neonate receives large doses of secretory Immunoglobulin A (sIgA) via breast milk; human breast milk has concentrations of sIgA 20–30 times higher than in gorilla or orangutan milk [44]. But eventually, the neonatal adaptive immune system must produce its own antibodies. The maternally inherited neonatal microbiome appears to have a crucial influence on the development of the immune system, both in its ability to combat pathogens but also in its ability to tolerate symbiotic and commensal microorganisms [38].

A final example is the association between breastfeeding and establishing a healthy neonatal gut microbiome. Breastfeeding behavior in the USA has improved, meeting the Healthy People 2020 goals [45] for initiation at over 80% and for exclusively breastfeeding for three months (46.9%), but falls just short of the targets for breastfeeding through 6 months and exclusively breastfeeding for 6 months [46]. The evidence for the benefits of breastfeeding over formula is well established and supported by fundamental mammalian reproductive adaptations relevant to child and maternal health. Milk is full of maternal immune cells, immune function molecules, hormones, cytokines and growth factors [47, 48]. This chemical signaling has played a critical role in all mammalian species in connecting mother and offspring during what we call the ‘fourth trimester’ in humans. Human milk also contains an extensive diversity of oligosaccharides, some of which have antimicrobial properties while others serve as prebiotics, providing food for beneficial microbes in the neonatal gut [48].

In addition, milk has been shown to harbor a distinct microbial community [49, 50]. One benefit of a coevolved microbiome is that it acts to reduce the ability of pathogenic organisms to colonize. This has led to several studies of the effectiveness of probiotics to treat and prevent mastitis in dairy cattle [51–54] and two published studies in women, one for treatment [55] and one for prevention of mastitis [56]. However, not all dairy cattle studies have shown a positive effect of probiotics [57], and both papers on women have been questioned concerning methodology and the appropriateness of the outcomes measured [58]. Despite the lack of evidence, providers may have to be ready to counsel patients regarding the use of probiotics for mastitis, as probiotic treatments for prevention and treatment of mastitis in women are already on the market [58]. Here an evolutionary perspective would counsel caution. We do not fully understand the function and effects of the milk microbiome, nor how it is established and maintained. Manipulations to modify it may have unintended consequences and cannot be assumed to be benign.

### Perception of the utility of evolution in medicine

The perception of the value of an evolutionary perspective in medical training appears to vary widely among physician–scientists and medical educators but is changing through time. In a 2003 survey of North American medical school deans, fewer than half of the participants believed that evolutionary biology was vital in the education of physicians [59]. Fewer than a third of schools were found to have covered at least half of the core topics in evolutionary biology, and even fewer schools had faculty with a PhD in evolutionary biology [59]. The main reasons offered for not adding evolution to the curriculum in medical schools were time constraints and a lack of faculty members who have the requisite expertise to teach the subject. Ten years after this initial survey of deans, a 2013 survey found that there has been an expanded teaching of evolution in medical schools, despite concerns of inciting controversy upon adding evolution to the curriculum [60].

Among the clinicians who responded to our survey, most indicted that an evolutionary perspective is important in medical research, public health, clinical medicine and medical education to varying degrees, and only a small subset (range 10.4–16.3%) responded this perspective is ‘not important’ (Table 2). For the specific examples in our survey, clinicians were most supportive of the importance of an evolutionary perspective in understanding antibiotic resistance (76.8%) and genetic carrier screening (71.8%). For other health care challenges, respondents generally considered an evolutionary perspective valuable for understanding the conditions, but fewer than half considered it to be important for clinical treatment (Table 3). Still, 34.1–49.3% considered an evolutionary perspective important or very important for clinical treatment of the health challenges listed in Table 3, and more of the responding obstetrician–gynecologists found value in an evolutionary perspective than answered it was not important for all but cervical cancer and sexually transmitted diseases (35.5% and 36.5% not important, respectively).

**Table 3.** Importance of an evolutionary perspective for understanding the following health care challenges versus clinical treatment of those challenges

Health care challenge	Important or very important for understanding	Important or very important for clinical treatment
Autoimmune diseases	62.7%	49.3%
Inherited thrombophilia	62.3%	48.6%
Gestational diabetes	56.6%	41.6%
Obstructed labor	51.4%	36.8%
Preterm labor/birth	50.0%	36.9%
Preeclampsia	48.1%	36.4%
Pelvic floor disorders	45.0%	34.7%
Cervical cancer	45.0%	34.1%
Sexually transmitted diseases	44.5%	35.6%

Studies support value of an evolutionary perspective when understanding and treating even these least supported health care challenges. The evolution of specific organs, such as the uterus and cervix, may play a role in the incidence of cancer [61], and, as mentioned before, the ‘mismatch’ between current lifestyles and the evolution of human birth could influence birth complications [13, 62]. Many sexually transmitted diseases have coevolved with humans; understanding their current and ancient global patterns requires an evolutionary perspective [63].

Regarding their own evolutionary knowledge, 41.8% of these providers said their knowledge of evolutionary medicine was ‘inadequate’ and they wish they knew more, while 45.5% said it was ‘adequate’ or ‘more than adequate’. Only 12.7% responded that evolutionary knowledge was unnecessary for clinical practice. There was no difference in how respondents rated their knowledge of evolutionary medicine based on years of practice (*F* = 0.012, *P* = 0.998).

Many medical schools do not make evolution a prerequisite for applying, nor do they place much value on evolution courses upon evaluating applications [60]. This is particularly concerning when our survey revealed most respondents’ primary time for evolution education was before medical school in undergraduate studies. Sixty-five percent (65.8%) of respondents reported they received ‘satisfactory’ or ‘comprehensive’ evolutionary biology education as undergraduates; however, only 26.8% and 12.3% of respondents found evolutionary education ‘satisfactory’ or ‘comprehensive’ in medical school and residency, respectively. If medical schools are not requiring evolution education before admittance, then the proportion of physicians well versed in evolutionary medicine will not increase at the rate we feel necessary. However, it is promising that respondents with fewer years of practice reported significantly more evolution education during their undergraduate years than respondents with more years of practice (*F* = 5.070, *P* = 0.002). We advocate that medical schools should require evolution coursework before enrolling to ensure medical students have at least a working knowledge of evolutionary principles [64].

Overall, physicians who completed our survey believe that knowledge of evolutionary medicine is important for students of their profession to know, but not important enough to change the status quo of current medical education programs. When considering devoting time to evolutionary biology in medical school, only 16.1% of respondents indicated that courses should be required. Eighty percent thought residency programs should incorporate evolutionary medicine, but only 34.1% thought it should be required. However, medical students may be more receptive to evolutionary medicine curriculum than respondents of our study; a study found that after completing an evolutionary medicine course, all students recommended the course for other students and rated the value of learning evolutionary medicine 4.7 out of 5 on a scale [65].

Our findings also highlight the importance of acknowledging political and religious biases when discussing evolutionary medicine. Politically conservative and more religious respondents had lower responses for the overall importance of evolution but rated their level of knowledge of evolutionary medicine no differently than respondents of other political and religious groups. This trend was more prominent if religion was reported to be important in providers’ clinical practice (*F* = 7.414, *P* = 0.001). On average, providers rated the importance of evolution in medicine as higher if they identified as having liberal political views (*F* = 12.821, *P* < 0.001). Wilson describes coursework that is effective in increasing interest and knowledge of evolution, even across religious spectrums, which could be a guiding framework for premed evolution courses and to incorporate instruction on evolutionary medicine in medical schools [66–68].

## Conclusion

There is support for including the evolutionary perspective in the training of providers of women’s health care from academics [14], medical researchers [69], medical educators [70], public health researchers [19] and based on our survey, clinicians. However, the difficulties of incorporating evolution into the crowded schedule of medical training are manifest. Placing a greater emphasis on evolution education at the undergraduate level as a condition of acceptance into medical school is a possible solution. However, we advocate offering courses on evolution at all levels of training. There are published materials that serve as resources for incorporating the evolutionary perspective into medical training, ranging from traditional textbooks [62, 71] to edited volumes presenting a variety of perspectives [72–74]. The growing awareness of the importance of the evolutionary perspective to understanding human health requires medical educators to confront the issue and to become creative in updating medical training to heighten knowledge of evolution.

## References

[1] Mayr E. The Growth of Biological Thought: Diversity, Evolution, and Inheritance. Cambridge: Harvard University Press, 1982.

[2] Dobzhansky T. Nothing in biology makes sense except in the light of evolution. Am Biol Teach 1973;35:125–9.

[3] Gluckman PD, Beedle AS, Hanson MA. Principles of Evolutionary Medicine. Oxford: Oxford University Press, 2009.

[4] Perlman RL. Evolution and Medicine. Oxford, UK: Oxford University Press, 2013.

[5] Harris EE, Malyango AA. Evolutionary explanations in medical and health profession courses: are you answering your students’ “why” questions? BMC Med Educ 2005;5:16–23. 15885137 10.1186/1472-6920-5-16 PMC1142319

[6] Wells JCK, Nesse RM, Sear R et al Evolutionary public health: introducing the concept. Lancet 2017;390:500–9. 28792412 10.1016/S0140-6736(17)30572-X

[7] Crosley EJ, Elliot MG, Christians JK *et al* Placental invasion, preeclampsia risk and adaptive molecular evolution at the origin of the great apes: evidence from genome-wide analyses. Placenta 2013;34:127–32. 23266291 10.1016/j.placenta.2012.12.001

[8] Committee on Obstetrics Practice, American College of Obstetricians and Gynecology, Committee Opinion 529 Placenta accretaspectrum. Obstet Gynecol 2018;132:e259.30461695

[9] US Preventative Service Task Force. Screening for preeclampsia US preventative services task force recommendation statement. JAMA 2017;317:1661–7. 28444286 10.1001/jama.2017.3439

[10] Haig D. Genetic conflicts in human pregnancy. Quart Rev Biol 1993;68:495–532. 8115596 10.1086/418300

[11] Arthur A, Terman A, Kurzontkowski C et al Molecular evolution of genes associated with preeclampsia: genetic conflict, antagonistic coevolution and signals of selection. J Evol Med 2018;6: 1–9.

[12] Elliot MG, Crespi BJ. Genetic recapulation of human pre-eclampsia risk during convergent evolution of reduced placental invasiveness in eutherian mammals. Philos Trans Soc B 2015;370:20140069. 10.1098/rstb.2014.0069 PMC4305170 25602073

[13] Rosenberg K, Trevathan W. Birth, obstetrics and human evolution. *Br J* Obstet Gynaecol 2002;109:1199–206. 10.1046/j.1471-0528.2002.00010.x 12452455

[14] Trevathan WR, Rosenberg KP. Evolutionary medicine and women’s reproductive health In:Schulkin J, Power ML (eds) Integrating Evolutionary Biology into Medical Education—for Maternal and Child Healthcare Students, Clinicians, and Scientists. Oxford: Oxford University Press, 2020.

[15] Kennell J, Klaus M, McGrath S et al Continuous emotional support during labor in a US hospital. JAMA 1991;265:2197–201. 2013951

[16] Pennisi E. Darwinian medicine’s drawn-out dawn. Science 2011;334:1486–7. 22174224 10.1126/science.334.6062.1486

[17] Tinbergen N. On aims and methods of ethology. Z Tierpsychol 2010;20:410–33.

[18] Nesse RM, Williams GC. Why we Get Sick: The New Science of Darwinian Medicine. New York: Times Books, 1994.

[19] Wells JCK. Evolutionary public health: how interventions may benefit from insights generated by life history theory. In: Schulkin J, Power ML (eds) Integrating Evolutionary Biology into Medical Education—for Maternal and Child Healthcare Students, Clinicians, and Scientists. Oxford: Oxford University Press, 2020.

[20] Paz S, Semenza JC. El Niño and climate change—contributing factors in the disoersal of Zika virus in the Americas? Lancet 2016; 387:745. 10.1016/S0140-6736(16)00256-7 26850984

[21] Graves JJL, Reiber C, Thanukos A et al Evolutionary science as a method to facilitate higher level thinking and reasoning in medical training. Evol Med Public Health 2016;2016:358–68. 27744353 10.1093/emph/eow029 PMC5101907

[22] Van Boeckel TP, Pires J, Silvester R et al Global trends in antimicrobial resistance in animals in low- and middle-income countries. Science 2019;365:eaaw1944. 31604207 10.1126/science.aaw1944

[23] CDC. Antibiotic Resistance Threats in the United States, 2013. Atlanta, GA: Centers for Disease Control & Prevention, 2013.

[24] Mannucci PM, Harari S, Martinelli I et al Effects on health of air pollution: a narrative review. Intern Emerg Med 2015;10:657–62. 26134027 10.1007/s11739-015-1276-7

[25] Rajagopalan S, Al-Kindi SG, Brook RD. Air Pollution and cardiovascular disease: JACC state-of-the-art review. J Am Coll Cardiol 2018;**72**:2054–70. 30336830 10.1016/j.jacc.2018.07.099

[26] Mendola P, Nobles C, Williams A et al Air pollution and preterm birth: do air pollution changes over time influence risk in consecutive pregnancies among low-risk women? Int J Environ Res Public Health 2019;16:3365. 10.3390/ijerph16183365 PMC6765877 31547235

[27] Zhang L, Liu W, Hou K et al Air pollution-induced missed abortion risk for pregnancies. Nat Sustain 2019;2:1011–7.

[28] American Public Health Association. Advancing a ‘One Health” approach to promote health at the human-animal-environment interface. Policy number 201712, 2017 https://www.apha.org/policies-and-advocacy/public-health-policy-statements/policy-database/2018/01/18/advancing-a-one-health-approach (accessed November 15, 2019).

[29] Kenyon C, Colebunders R, Crucitti T. The global epidemiology of bacterial vaginosis: a systematic review. Am J Obstet Gynecol 2013;209:505–23. 23659989 10.1016/j.ajog.2013.05.006

[30] Nasioudis D, Linhares IM, Ledger WJ *et al* Bacterial vaginosis: a critical analysis of current knowledge. *Br J* Obstet Gynaecol 2017;124:61–9. 10.1111/1471-0528.14209 27396541

[31] Brotman RM. Vaginal microbiome and sexually transmitted infections: an epidemiologic perspective. J Clin Invest 2011;121:4610–7. 22133886 10.1172/JCI57172 PMC3225992

[32] Amsel R, Totten PA, Spiegel CA et al Nonspecific vaginitis. Diagnostic criteria and microbial and epidemiologic associations. Am J Med 1983;74:14–22. 6600371 10.1016/0002-9343(83)91112-9

[33] Nugent RP, Krohn MA, Hillier SL. Reliability of diagnosing bacterial vaginosis is improved by a standardized method of gram stain interpretation. J Clin Microbiol 1991;29:297–301. 1706728 10.1128/jcm.29.2.297-301.1991 PMC269757

[34] Ravel J, Gajer P, Abdo Z et al Vaginal microbiome of reproductive-age women. Proc Natl Acad Sci USA 2011;108:4680–7. 20534435 10.1073/pnas.1002611107 PMC3063603

[35] Ma B, Forney LJ, Ravel J. The vaginal microbiome: rethinking health and diseases. Ann Rev Microbiol 2012;66:371–89. 22746335 10.1146/annurev-micro-092611-150157 PMC3780402

[36] Lamont RF, Sobel JD, Akins RA et al The vaginal microbiome: new information about genital tract flora using molecular based techniques. *Br J* Obstet Gynaecol 2011;118:533–49. 10.1111/j.1471-0528.2010.02840.x PMC3055920 21251190

[37] Yildirim S, Yeoman C, Janga S et al Primate vaginal microbiomes exhibit species specificity without universal *Lactobacillus* dominance. ISME J 2014;8:2431–44. 25036926 10.1038/ismej.2014.90 PMC4260710

[38] McDonald B, McCoy K. Maternal microbiota in pregnancy and early life: the maternal microbiota shape offspring development, including susceptibility to some illnesses. Microbiology 2019; 365:984–5. 10.1126/science.aay0618 31488675

[39] https://www.cdc.gov/nchs/fastats/delivery.htm (22 November 2019, date last accessed).

[40] Montoya-Williams D, Lemas DJ et al The neonatal microbiome and its partial role in mediating the association between birth by cesarean section and adverse pediatric outcomes. Neonatology 2018;114:103–11. 29788027 10.1159/000487102 PMC6532636

[41] Dominguez-Bello MG, De Jesus-Laboy KM, Shen N et al Partial restoration of the microbiota of cesarean-born infants via vaginal microbial transfer. Nat Med 2016;22:250–3. 26828196 10.1038/nm.4039 PMC5062956

[42] Committee on Obstetric Practice, American College of Obstetricians and Gynecology, Committee Opinion 725 Vaginal seeding. Obstet Gynecol 2017;130:e274–8. 29064974 10.1097/AOG.0000000000002402

[43] Coe CL, Lubach GL, Izard KM. Progressive improvement in the transfer of maternal antibody across the order primates. Am J Primatol 1994;32:51–5. 31936907 10.1002/ajp.1350320106

[44] Garcia M, Power ML, Moyes KM. Immuniglobulin A and nutrients in milk from great apes throughout lactation. Am J Primatol 2017;79:e22614. 10.1002/ajp.22614 28118501

[45] https://www.healthypeople.gov/ (22 November 2019, date last accessed).

[46] https://www.cdc.gov/breastfeeding/data/reportcard.htm (22 November 2019, date last accessed).

[47] Power ML, Schulkin J. Maternal regulation of offspring development in mammals is an ancient adaptation tied to lactation. Appl Transl Genom 2013;**2**:55–63. 27896056 10.1016/j.atg.2013.06.001 PMC5121250

[48] Power ML, Schulkin J. Milk: The Biology of Lactation. Baltimore: Johns Hopkins University Press, 2016.

[49] Cabrera-Rubio R, Collado MC, Laitinen K et al The human milk microbiome changes over lactation and is shaped by maternal weight and mode of delivery. Am J Clin Nutr 2012;96:544–51. 22836031 10.3945/ajcn.112.037382

[50] Muletz-Wolz CR, Kurata NP, Himschoot EA et al Diversity and temporal dynamics of primate milk microbiomes. Am J Primatol 2019;81:e22994. 10.1002/ajp.22994 PMC6842035 31219214

[51] Klostermann K, Crispie F, Flynn J et al Intramammary infusion of a live culture of *Lactobacillus lactis* for treatment of bovine mastitis; comparison with antibiotic in field trials. J Dairy Res 2008;75:365–73. 18680622 10.1017/S0022029908003373

[52] Soleimani NA, Kermanshahi RK, Yakhchali B *et al* Antagonistic activity of probiotic lactobacilli against *Staphylococcus aureus* isolated from bovine mastitis. Afr J Microbiol Res 2010; 4:2169–73.

[53] Espeche MC, Pellegrino M, Frola I et al Lactic acid bacteria from raw milk as potentially beneficial strains to prevent bovine mastitis. Anaerobe 2012;18:103–9. 22261519 10.1016/j.anaerobe.2012.01.002

[54] Bouchard DS, Seridan B, Seridan B et al Lactic acid bacteria isolated from bovine mammary microbiota: potential allies against bovine mastitis. PLoS One 2015;10:e0144831. 26713450 10.1371/journal.pone.0144831 PMC4694705

[55] Arroyo R, Martín V, Maldonado A et al Treatment of infectious mastitis during lactation: antibodies versus oral administration of Lactobacilli isolated from breast milk. Clin Infect Dis 2010;50:1551–73. 20455694 10.1086/652763

[56] Fernández L, Cardenas N, Arroyo R et al Prevention of infectious mastitis by oral administration of *Lactobacillus salivarius* PS2 during late pregnancy. Clin Infect Dis 2016;62:568–73. 26611780 10.1093/cid/civ974

[57] Greene WA, Gano AM, Smith KL et al Comparison of probiotic and antibiotic intrammary therapy of cattle with elevated somatic cell counts. J Dairy Sci 1991;74:2976–81. 1779053 10.3168/jds.S0022-0302(91)78483-X

[58] Amir LH, Griffin L, Cullinane M *et al* Probiotics and mastitis: evidence-based marketing? Int Breastfeed J 2016;11:19. 27446229 10.1186/s13006-016-0078-5 PMC4955247

[59] Nesse RM, Schiffman JD. Evolutionary biology in the medical school curriculum. BioScience 2003;53:585–7.

[60] Hidaka BH, Asghar A, Aktipis CA et al The status of evolutionary medicine education in North American medical schools. BMC Med Educ 2015;15:38–47. 25884843 10.1186/s12909-015-0322-5 PMC4355969

[61] Thomas F, Nesse RM, Gatenby R et al Evolutionary ecology of organs: a missing link in cancer development? Trends Cancer 2016;2:409–15. 28741494 10.1016/j.trecan.2016.06.009

[62] Gluckman P, Beedle A, Buklijas T et al Principles of Evolutionary Medicine, 2nd edn Oxford: University Press, 2016.

[63] Pimenoff V, Houldcroft C, Rifkin R *et al* The role of aDNA in understanding the coevolutionary patterns of human sexually transmitted infections. Genes 2018;9:317. 10.3390/genes9070317 PMC6070984 29941858

[64] Labov JB. Evolutionary medicine and the medical school curriculum: meeting students along their paths to medical school. Evo Edu Outreach 2011;4:561–6.

[65] Abbott A, Abboud G. Evolutionary medicine: a model for medical school introduction. Med Educ 2006;40:471–89. 16635141 10.1111/j.1365-2929.2006.02428.x

[66] Wilson DS. Evolution for everyone: how to increase acceptance of, interest in, and knowledge about evolution. PLoS Biol 2005;3:e364. 16336048 10.1371/journal.pbio.0030364 PMC1311567

[67] Barnes ME, Brownell SE. Practices and perspectives of college instructors on addressing religious beliefs when teaching evolution. CBE Life Sci Educ 2016;15:1–19. 10.1187/cbe.15-11-0243 PMC4909340 27193289

[68] Barnes ME, Brownell SE, A call to use cultural competence when teaching evolution to religious college students: introducing religious cultural competence in evolution education (ReCCEE). CBE Life Sci Educ 2017;1. 10.1187/cbe.17-04-0062 PMC5749973 29167225

[69] Gluckman PD, Low FM, Hanson MA. Evolutionary medicine, pregnancy, and the mismatch pathways to increased disease risk. In:Schulkin J, Power ML (eds) Integrating Evolutionary Biology into Medical Education—for Maternal and Child Healthcare Students, Clinicians, and Scientists. Oxford: Oxford University Press, 2020.

[70] Stuebe AM, Tully KP. Optimizing maternal-infant health: ameliorating the mismatch between evolved dyadic needs and the current culture of health around the 4th trimester. In: Schulkin J, Power ML (eds). Integrating Evolutionary Biology into Medical Education—for Maternal and Child Healthcare Students, Clinicians, and Scientists. Oxford: Oxford University Press, 2020.

[71] Stearns SC, Medzhitov R. Evolutionary Medicine. Oxford: Oxford University Press, 2015.

[72] Trevathan WR, Smith EO, McKenna JJ. Evolutionary Medicine. New York: Oxford University Press, 1999.

[73] Stearns SC, Koella JC. Evolution in Health and Disease. Oxford: Oxford University Press, 2008.

[74] Schulkin J, Power ML (eds) Integrating Evolutionary Biology into Medical Education—for Maternal and Child Healthcare Students, Clinicians, and Scientists. Oxford: Oxford University Press, 2020.

